# Optimal cumulative dose of cisplatin for concurrent chemoradiotherapy among patients with non-metastatic nasopharyngeal carcinoma: a multicenter analysis in Thailand

**DOI:** 10.1186/s12885-020-07024-8

**Published:** 2020-06-03

**Authors:** Nuttapong Ngamphaiboon, Arunee Dechaphunkul, Jiraporn Setakornnukul, Tanadech Dechaphunkul, Rungarun Jiratrachu, Bhoom Suktitipat, Chuleeporn Jiarpinitnun, Poompis Pattaranutaporn, Pongwut Danchaivijitr

**Affiliations:** 1 Division of Medical Oncology, Department of Medicine, Faculty of Medicine, Ramathibodi Hospital, Mahidol University, 270 Rama VI Road, Ratchathewi, Bangkok, 10400 Thailand; 2 Division of Medical Oncology, Department of Medicine, Faculty of Medicine, Songklanagarind Hospital, Prince of Songkla University, Hat Yai, Thailand; 3 Division of Radiation Oncology, Department of Radiology, Faculty of Medicine, Siriraj Hospital, Mahidol University, Bangkok, Thailand; 4 Department of Otorhinolaryngology Head and Neck Surgery, Faculty of Medicine, Songklanagarind Hospital, Prince of Songkla University, Hat Yai, Thailand; 5 Division of Radiation Oncology, Department of Radiology, Faculty of Medicine, Songklanagarind Hospital, Prince of Songkla University, Hat Yai, Thailand; 6 Department of Biochemistry, Faculty of Medicine, Siriraj Hospital, Mahidol University, Bangkok, Thailand; 7 Integrative Computaional BioScience Center, Mahidol University, Bangkok, Thailand; 8 Division of Radiation Oncology, Department of Radiology, Faculty of Medicine, Ramathibodi Hospital, Mahidol University, Bangkok, Thailand; 9 Division of Medical Oncology, Department of Medicine, Faculty of Medicine Siriraj Hospital, Mahidol University, 2 Thanon Wang Lang, Siriraj, Bangkok Noi, Bangkok, 10700 Thailand

**Keywords:** Nasopharyngeal carcinoma, Cisplatin, Chemoradiotherapy, Cumulative dose, Nephrotoxicity

## Abstract

**Background:**

Chemoradiotherapy (CRT) with high cumulative doses (CDs) of cisplatin has been considered the standard of care for non-metastatic nasopharyngeal carcinoma (NPC). However, given most patients’ inability to tolerate high CDs due to cisplatin-related toxicities, the optimal CD of cisplatin during CRT remains undetermined.

**Methods:**

Patients with non-metastatic NPC who received CRT with cisplatin between 2007 and 2017 were identified through the Thai head and neck cancer multicenter database and then categorized according to cisplatin CD (mg/m^2^) received. All complications and cisplatin-related toxicities during CRT were recorded.

**Results:**

We identified 779 non-metastatic NPC patients receiving low (≤150; *n* = 97), intermediate (151–250; *n* = 411), and high (> 250; *n* = 271) CDs of cisplatin. Low CD patients had significantly lower mean actual radiation dose (*p* < 0.001) and more radiotherapy delay (*p* = 0.010), while intermediate CD patients had the least hospitalization (*p* < 0.001). Overall, 39.3% of the patients experienced cisplatin-related toxicity, which was associated with poor overall survival (OS) (*p* = 0.001). Acute kidney injury was observed in 7% in all patients, which was highest among low CD patients (15.5%; *p* = 0.002). Intermediate CD patients had significantly longer median OS than the low and high groups (64 vs. 49.8 vs. 53.2, respectively; *p* = 0.015). Univariate, but not multivariate, analysis showed that CD of cisplatin was significantly associated with OS.

**Conclusion:**

CD of cisplatin during CRT was not an independent prognostic factor for OS. An intermediate CD induced minimal toxicity without compromising survival and should be considered the optimal CD. Nonetheless, a randomized phase 3 study evaluating the optimal CD of cisplatin is warranted.

## Background

Nasopharyngeal carcinoma (NPC) is highly endemic to Southern China, Maghreb region of North Africa, parts of the Middle East, and Southeast Asia, including Thailand [1, 2]. Epstein–Barr virus (EBV) plays a major role in carcinogenesis. Ninety-six percent of Thai patients with NPC are associated with EBV [3]. Concurrent chemoradiotherapy (CRT) with cisplatin is the standard of care for local advanced NPC treatment [4]. Several phase III clinical studies utilized high-dose cisplatin, either 100 mg/m^2^ every 3 weeks for three cycles [cumulative dose (CD), 300 mg/m^2^] or 40 mg/m^2^ weekly for seven cycles (CD, 280 mg/m^2^), for using concurrently with radiotherapy (RT) [5–8]. However, no study has evaluated the optimal cisplatin CD administration during CRT. A post-hoc analysis from a Chinese prospective phase III study of patients with non-metastatic NPC concurrently receiving weekly cisplatin and radiotherapy (RT) reported a median cisplatin CD of 240 mg/m^2^, despite protocols requiring a CD of 280 mg/m^2^ [9]. Several retrospective studies demonstrated real-world data that majority of patients received less than the standard recommendations for cisplatin CD (280–300 mg/m^2^) [8, 10–12]. However, patients receiving a lower cisplatin CD had comparable survival with patients receiving the recommended CD. Thus, the lowest efficacious cisplatin CD during CRT remains unidentified.

About 13.6% patients with cancer treated with cisplatin develop nephrotoxicity, i.e., acute kidney injury (AKI), which is significantly associated with administrated cisplatin dose [13]. Incidences of nephrotoxicity among head and neck cancer patients receiving cisplatin CRT were 30–34%, higher than in those with other cancer types [14, 15]. Therefore, we evaluated the optimal cisplatin CD for definitive CRT that would maintain efficacy and minimize toxicity among patients with non-metastatic NPC in a multicenter setting in Thailand.

## Methods

### Patient population

We identified patients with histologically confirmed non-metastatic NPC who received treatment at the three largest cancer centers in Thailand, Ramathibodi and Siriraj Hospitals, Mahidol University, and Songklanagarind Hospital, Prince of Songkla University, between 2007 and 2017. All medical records were retrospectively reviewed. Patient characteristics, treatments, toxicities, complications, and survival rates were abstracted and recorded in the common electronic case record form of the large multicenter multidisciplinary database for Thai head and neck cancer patients using the REDCap platform. This database was established in 2016 and was funded by the Thailand Grand Challenge Program for Research University Network (RUN) under the Precision Medicine for Cancer project. The present study was approved by the Institutional Ethic Committee.

Patients with non-metastatic NPC who received cisplatin for concurrent CRT were identified from the database. Those who received induction chemotherapy, had distant metastasis at diagnosis, or received non-cisplatin chemotherapy for CRT were excluded. For patients who cisplatin was initially started and subsequently terminated during CRT from any reasons, substitution of concurrent carboplatin CRT was allowed at their physician’s discretion. These patients were included in this analysis. All patients in this study must be treated with definitive CRT with the planned total dose of radiation of 7000 cGy. Radiation technique including 2D, 3D, and IMRT was selected for each patent based on their treating physicians according to standard of care, availability, and physician’s discretion at the time of treatment. Adjuvant platinum doublet chemotherapy was given after completion of CRT following local standard practice at treating physician’s discretion. Overall survival (OS), recurrence-free survival (RFS), distant recurrence-free survival (DRFS), and locoregional recurrence-free survival (LRFS) were determined. Death status was validated and confirmed through the Thai Social Security Death Index database.

### Cisplatin dose for chemoradiotherapy and its complications

CD of cisplatin (mg/m^2^) used during CRT was calculated and classified into low, intermediate, and high. The dose cutoffs were identified using the area under the curve (AUC) associated with the gold standard for predicting death status. The upper cutoff of 250 mg/m^2^ was selected according to the AUC with significance. The CD of 150 mg/m^2^ was selected for the lower cutoff since the low CD of cisplatin during CRT was previously reported ranging between 100 and 160 mg/m^2^ in the literature [10, 16, 17]. Thus, CD of cisplatin during CRT in this study was categorized into low (≤150 mg/m^2^; *n* = 97), intermediate (151–250 mg/m^2^; *n* = 411), and high (> 250 mg/m^2^; *n* = 271; Supplement 1). The correlation between aforementioned dose levels and patient characteristics, RT treatment, treatment tolerability, complications, and survival was then determined.

Acute complications during CRT were defined as experience of any cisplatin or RT interruption, treatment delay (> 7 days), hospitalization (> 24 h), and/or discontinuation of cisplatin or RT. Cisplatin-related complications included creatinine clearance (CCr) decline after CRT (calculated using the Cockcroft–Gault formula), AKI [18], acute kidney disease (AKD), defined by (i) GFR < 60 ml/min/1.73 m^2^ for < 3 months, or (ii) decrease in GFR by ≥35%, or (iii) increase in serum creatinine by > 50% for < 3 months [19], electrolyte imbalance requiring hospitalization during CRT, and ototoxicity.

### Statistical analysis

All analyses were performed using the STATA/MP 14.1. Descriptive statistics, including mean ± SD or median (range), were used for continuous variables. Differences between the three categories were compared using one-way analysis of variance (normal distribution) or Kruskal–Wallis test (non-normal distribution). Significant differences in proportions were determined using the Chi-square or Fisher’s exact test, as appropriate. Receiver operating characteristic analysis was conducted to determine the cutoff CD of cisplatin (mg/m^2^) using the gold standard for predicting death status. Associated risk factors were initially screened using univariate and multivariate Cox regression analyses for OS with significance set at *p* < 0.10. Identified variables were subsequently assessed using backward stepwise regression with significance set at *p* ≤ 0.05. The final model included cisplatin CD during CRT and other significant factors. The goodness-of-fit assumption was assessed using the Hosmer–Lemeshow method with significance set at *p* > 0.05. Cumulative hazard curves were modeled using the Kaplan–Meier method and compared using the Log-rank test. A *p* value of < 0.05 indicated statistical significance.

## Results

### Patient characteristics

Overall 779 eligible patients with non-metastatic NPC were identified from the database. Baseline patient and pathological characteristics according to CD of cisplatin are summarized in Table 1. Majority of the patients in this cohort (99%) had WHO grades II and III disease. Approximately 50% patients underwent prophylactic feeding tube placement prior to CRT. Among those with a prophylactic feeding tube, 88% underwent percutaneous endoscopic gastrostomy, whereas 12% underwent nasogastric tube insertion. Patients who received low CD of cisplatin were significantly older at diagnosis (*p* < 0.001) and had poorer smoking status (*p* = 0.003), lower LN stage (*p* = 0.016), higher incidences of comorbidities, including cardiac disease (*p* = 0.009), diabetes mellitus (*p* = 0.014), and hypertension (*p* = 0.043), and higher baseline CCr (*p* < 0.001) than other groups.

**Table 1:** Patient characteristics

Patient characteristics N (%)	Low dose 97 (12.4%)	Intermediate dose 411 (52.8%)	High dose 271 (34.8%)	***P*** value
Median age (range) years	54.5 [16–78]	51 [19–77]	46 [16–73]	< 0.001
Age ≥ 65 years	22 (22.9%)	48 (11.9%)	11 (4.2%)	< 0.001
Sex				
Female	29 (29.9%)	137 (33.3%)	74 (27.3%)	0.243
Male	68 (70.1%)	274 (66.7%)	197 (72.7%)	
Smoking status				
Never	48 (52.2%)	261 (67.8%)	146 (57%)	0.003
Ever	44 (47.8%)	124 (32.2%)	110 (43%)	
Median pack-year (range)	14 (7.13, 26.75)	15 (5, 25)	14.13 (5.25, 22)	0.946
ECOG status				
0–1	57 (95%)	225 (98.7%)	182 (98.4%)	0.167
≥ 2	3 (5%)	3 (1.3%)	3 (1.6%)	
Baseline mean BMI (±SD)	23.37 ± 4.2	23.47 ± 4.15	23.37 ± 4.17	0.952
< 18.5	12 (12.4%)	25 (6.1%)	16 (6%)	0.199
18.5–22.9	37 (38.1%)	181 (44.3%)	123 (45.9%)	
≥ 23	48 (49.5%)	203 (49.6%)	129 (48.1%)	
WHO classification				
I	0 (0%)	3 (0.7%)	7 (2.7%)	0.147
II	57 (58.8%)	217 (54%)	138 (53.7%)	
III	40 (41.2%)	182 (45.3%)	112 (43.6%)	
T-stage				
T1–2	23 (23.7%)	96 (23.4%)	50 (18.5%)	0.156
T3–4	73 (75.3%)	305 (74.2%)	208 (76.8%)	
Tx	1 (1%)	10 (2.4%)	13 (4.8%)	
LN Stage				
0–1	34 (35.1%)	140 (34.1%)	71 (26.2%)	0.016
2–3	63 (64.9%)	263 (64%)	187 (69%)	
Nx	0 (0%)	8 (1.9%)	13 (4.8%)	
Stage at diagnosis				
I	0 (0%)	1 (0.2%)	1 (0.4%)	0.029
II	18 (18.6%)	70 (17%)	38 (14%)	
III	36 (37.1%)	190 (46.2%)	124 (45.8%)	
IVa	28 (28.9%)	104 (25.3%)	54 (19.9%)	
IVb	15 (15.5%)	38 (9.2%)	41 (15.1%)	
Unknown	0 (0%)	8 (1.9%)	13 (4.8%)	
Any comorbidity	30 (30.9%)	92 (22.4%)	57 (21%)	0.127
Cardiac	6 (6.2%)	10 (2.4%)	2 (0.7%)	0.009
Diabetes	14 (14.4%)	37 (9%)	14 (5.2%)	0.014
Hyperlipidemia	7 (7.2%)	20 (4.9%)	16 (5.9%)	0.622
Hypertension	22 (22.7%)	68 (16.5%)	33 (12.2%)	0.043
Kidney Disease	0 (0%)	3 (0.7%)	0 (0%)	0.260
Mean Baseline CCr (±SD)	81 ± 27	92 ± 28	129 ± 29	< 0.001
Prophylactic feeding tube	48 (49.5%)	201 (48.9%)	152 (56.1%)	0.169

*ECOG* Eastern Cooperative Oncology Group, *BMI* body mass index, *CCr* creatinine clearance

### Treatments and acute complications during chemoradiotherapy

#### Chemotherapy

The mean CD of low-, intermediate-, and high-dose groups were 104, 207, and 287 mg/m^2^, respectively (Table 2). The intermediate CD group received more 3-weekly regimens than the low and high CD groups (*p* = 0.031). Most patients in the high CD group (98.5%) completed the planned cisplatin cycle. Only 60 patients in the low CD group (62%) received adjuvant chemotherapy, whereas 363 (88%) and 252 (93%) patients in the intermediate and high CD groups received the same, respectively (*p* < 0.001). However, the high CD group had a significantly lower mean CD of adjuvant cisplatin than the other groups (*p* = 0.002). Patients treated with low and intermediate CD were able to complete 3 cycles of adjuvant chemotherapy significantly more than high CD group (71.2% vs 74.3% vs 56.1%; *p* < 0.001).

**Table 2:** Treatment and acute complications during chemoradiotherapy

Treatment N (%)	Low dose 97 (12.4%)	Intermediate dose 411 (52.8%)	High dose 271 (34.8%)	***P*** value
**Chemotherapy during CRT**				
Mean cumulative dose of cisplatin during CRT (mg/m^2^) (±SD)	103.62 ± 27.94	206.9 ± 23.99	286.54 ± 16.03	< 0.001
CRT regimen				
Q1 week	11 (11.3%)	26 (6.3%)	32 (11.8%)	0.031
Q3 week	86 (88.7%)	385 (93.7%)	239 (88.2%)	
Completed planned cycle during CRT	1 (1%)	154 (37.5%)	267 (98.5%)	< 0.001
Median number of actual given cycles during CRT (range)				
Weekly	3 (1, 3)	6 (5, 6)	7 (7, 7)	< 0.001
Q3 week	1 (1, 2)	2 (2, 3)	3 (3, 3)	< 0.001
Adjuvant chemotherapy regimen				
Cisplatin-5FU	36 (37.1%)	345 (83.9%)	230 (84.9%)	< 0.001
Carboplatin-5FU	24 (24.7%)	18 (4.4%)	22 (8.1%)	
No adjuvant chemotherapy	37 (38.1%)	48 (11.7%)	19 (7%)	
Number of completed cycle of adjuvant chemotherapy				
1 cycle	7 (11.9%)	38 (10.3%)	25 (10.2%)	< 0.001
2 cycles	10 (16.9%)	57 (15.4%)	83 (33.7%)	
3 cycles	42 (71.2%)	274 (74.3%)	138 (56.1%)	
Mean cumulative dose of adjuvant cisplatin (mg/m^2^) (±SD)	199.24 ± 93.3	203.67 ± 63.45	184.08 ± 64.24	0.002
**Radiotherapy (RT)**				
Technique				
IMRT	61 (62.9%)	298 (72.5%)	134 (49.4%)	< 0.001
3D	16 (16.5%)	51 (12.4%)	55 (20.3%)	
2D	19 (19.6%)	53 (12.9%)	77 (28.4%)	
Unknown	1 (1%)	9 (2.2%)	5 (1.8%)	
Mean actual dose (cGy) (±SD)	6563.75 ± 1399.55	6974.26 ± 346.24	6989.42 ± 181.96	< 0.001
Median duration (weeks) (range)	7.07 (6.57,8.14)	7.14 (6.71,7.86)	7.29 (7.0,8.0)	0.013
RT delay (> 7 days)	28 (29.2%)	67 (16.7%)	62 (23.4%)	0.010
**Acute complication during CRT**				
Cisplatin Interruption	64 (66%)	186 (45.3%)	111 (41%)	< 0.001
Cisplatin Delay	35 (36.1%)	75 (18.2%)	43 (15.9%)	< 0.001
Hospitalization	11 (11.5%)	15 (3.7%)	30 (11.1%)	< 0.001
Termination of cisplatin	66 (68%)	81 (19.7%)	13 (4.8%)	< 0.001
- Switch to carboplatin	31 (45.5%)	8 (9.9%)	0 (0%)	< 0.001
At least 1 acute complication	77 (79.4%)	191 (46.5%)	123 (45.4%)	< 0.001
**Cisplatin-related toxicity during CRT**				
All cisplatin-related toxicity	49 (50.5%)	144 (35%)	113 (41.7%)	0.012
Mean percent dropped of CCr after CRT completion (±SD)	−8.79 ± 40.09	−18.23 ± 26.6	−24.03 ± 24.68	0.001
AKI	15 (15.5%)	25 (6.1%)	14 (5.2%)	0.002
AKD	27 (39.1%)	98 (28.7%)	81 (38.2%)	0.038
Hospitalization due to cisplatin	10 (10.3%)	10 (2.4%)	23 (8.5%)	< 0.001
Treatment interruption due to cisplatin	36 (37.1%)	71 (17.3%)	41 (15.1%)	< 0.001

*CRT* chemoradiotherapy, *5FU* Fluorouracil, *IMRT* intensity-modulated radiation therapy, *AKI* acute kidney injury, *AKD* acute kidney disease

#### Radiotherapy

Overall, 493 patients (63%) concurrently received intensity-modulated radiation therapy (IMRT) with cisplatin as the definitive treatment, whereas 122 (16%) and 149 (19%) patients underwent 3D and 2D techniques, respectively. More patients in the intermediate CD group (72.5%) received IMRT than the low (63%) and high CD (49%) groups (*p* < 0.001; Table 2). Patients treated with IMRT technique had significant better smoking status, ECOG performance status, higher baseline BMI, earlier stage at diagnosis, but more comorbidities including diabetes, hyperlipidemia, and hypertension. (Supplement 2) Moreover, IMRT patients had significantly higher baseline CCr, less prophylactic feeding tube, and less CD of cisplatin during CRT. Only12 patients in the 2D group (11.4%) did not receive adjuvant chemotherapy when compared with 7 (5.7%) and 76 (15.4%) patients in the 3D and IMRT groups, respectively (*p* = 0.002). However, those receiving a low CD of cisplatin had a significantly low mean actual RT dose (*p* < 0.001). Patients in the high CD group had longer duration of RT (*p* = 0.013), whereas RT delay was more common in low CD patients (*p* = 0.010).

#### Acute complication and cisplatin-related toxicity

Patients receiving a low CD of cisplatin experienced higher rates of cisplatin interruption, delay, and termination of cisplatin (*p* < 0.001) compared with the intermediate and high CD groups (Table 2). Intermediate CD patients had the lowest incidence (4%) of hospitalization during CRT (*p* < 0.001). Among those, in whom cisplatin was terminated, 31 (45.5%) and 8 (9.9%) in the low and intermediate CD groups subsequently received concurrent carboplatin and radiation, respectively. The low CD group had more patients with at least one acute complication (79.4%) than the other groups.

Overall, cisplatin-related toxicity occurred in 306 patients (39.3%), with the low CD group having the highest incidence (50.5%; Table 2). The most common cisplatin-related toxicities included nephrotoxicity (26.4%), grade 3–4 vomiting (4%), grade 3–4 electrolyte imbalance (3.6%), and grade 3–4 infection (including febrile neutropenia; 3.3%; Supplement 3)**.** Mean percent drop in CCr was significantly associated with higher CD of cisplatin (*p* = 0.001). Overall, 7% patients developed AKI during CRT, with the low CD group having the highest number (15.5%; *p* = 0.002). Furthermore, the incidence of AKD was comparable between the high (38.2%) and low (39.1%) cisplatin groups but lower in the intermediate group (28.7%; *p* = 0.038).

#### Survival

**Fig. 1:**
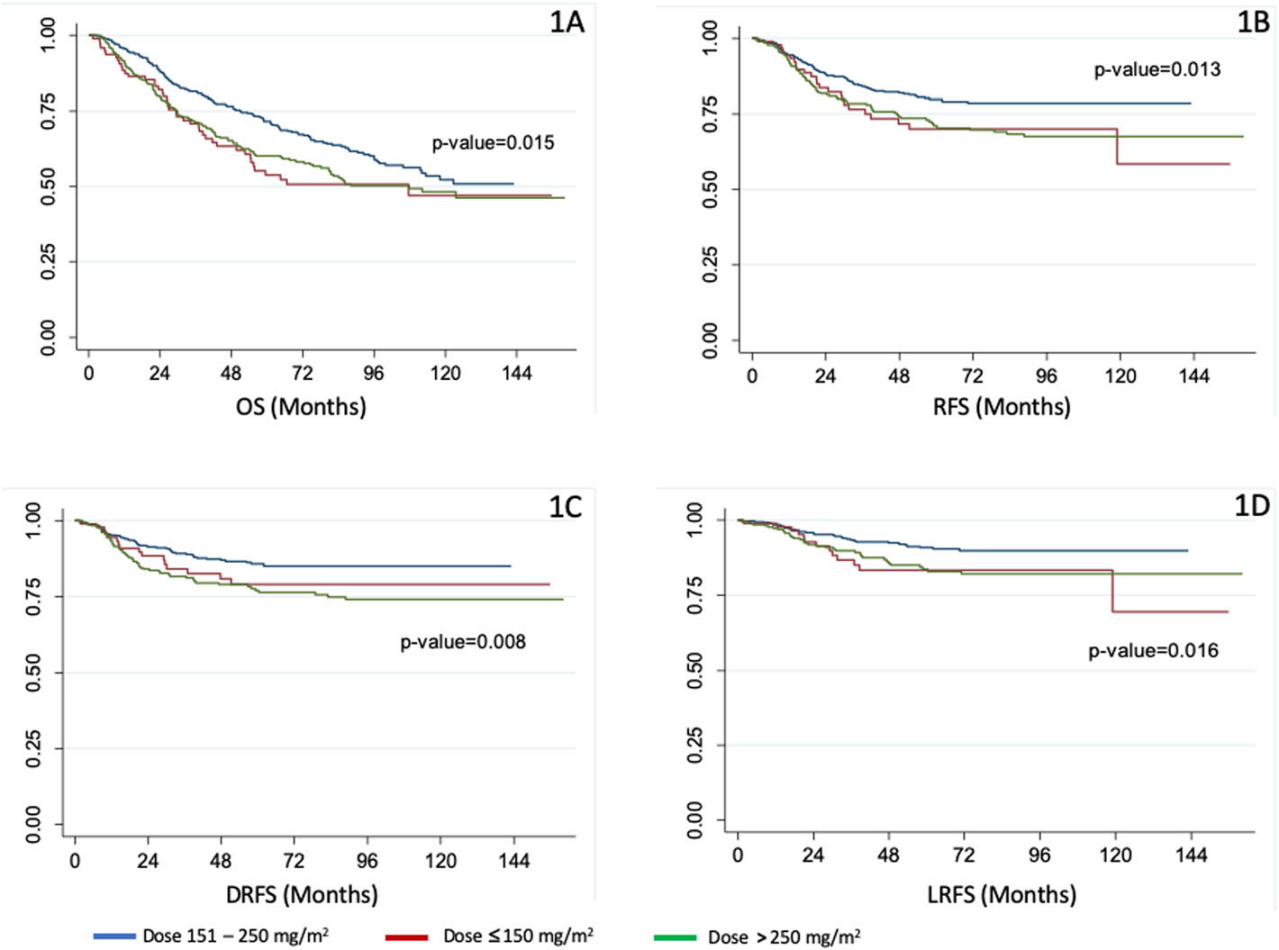
Patient survival based on cumulative dose of cisplatin (mg/m^2^) during chemoradiotherapy. **a** Overall survival (OS), **b** Recurrence-free survival (RFS), **c** distant recurrence-free survival (DRFS), **d** Locoregional recurrence-free survival (LRFS)

**Fig. 2:**
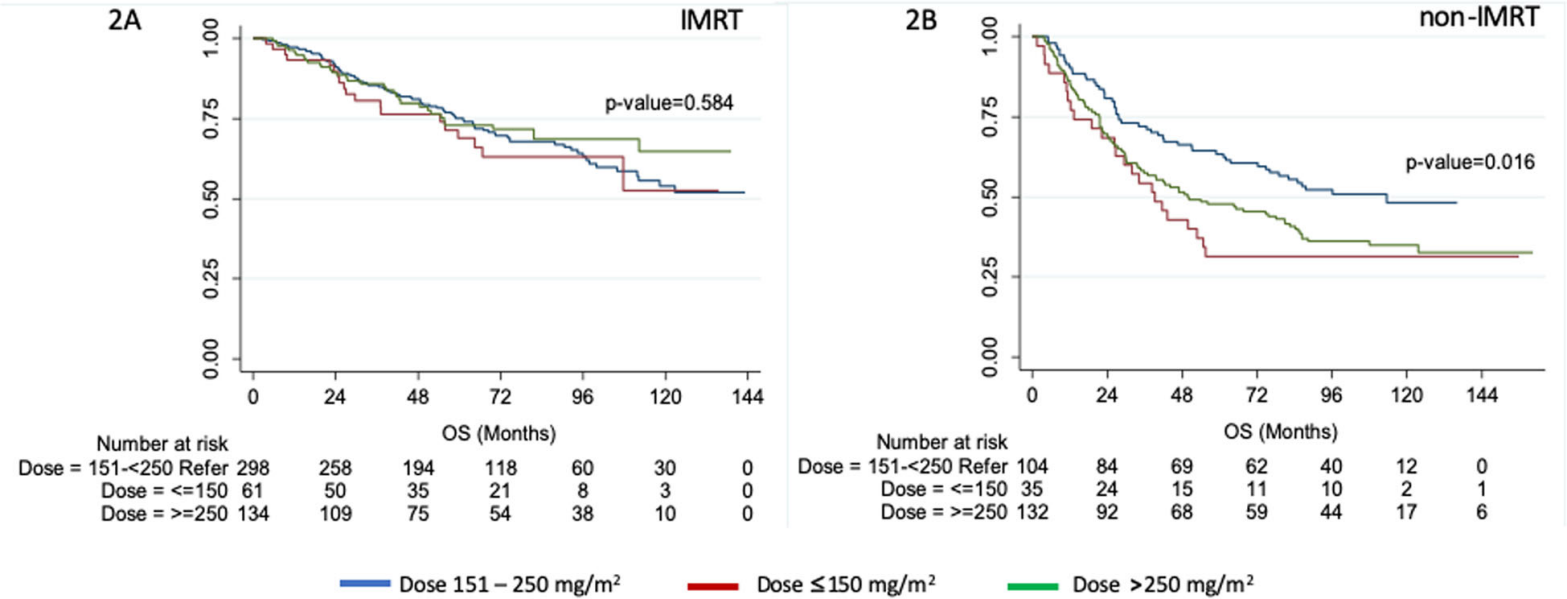
Overall survival using the RT technique. **a** intensity-modulated radiation therapy, **b** non-intensity-modulated radiation therapy

The median follow-up duration of the study was 59.4 months. Patients who received an intermediate CD of cisplatin had significantly longer median OS (64 months) than low and high (49.8 and 53.2 months, respectively) CD groups (*p* = 0.015; Fig. 1a). Moreover, 5-year OS among patients treated with low, intermediate, and high CD of cisplatin were 54, 72, and 60%, respectively (*p* = 0.004). The intermediate CD group (64%) had a significantly higher 7-year OS than the low (51%) and high (53%) CD groups (*p* < 0.001). Subset analysis of patients who underwent IMRT showed no significant differences in OS among the cisplatin CD groups (*p* = 0.584), whereas intermediate CD patients who underwent non-IMRT had significantly longer OS than the other groups (*p* = 0.016; Fig. 2). Those who experienced cisplatin-related toxicity during CRT had significantly shorter OS (*p* = 0.001; Supplement 4). In addition, the intermediate CD patients had significantly longer RFS, DRFS, and LRFS than the low- and high-dose groups (Fig. 1b–d).

Univariate and multivariate analyses of OS are summarized in Table 3. Univariate, but not multivariate, analysis showed that CD of cisplatin was significantly associated with OS. Multivariate analysis showed that age ≥ 65, stage IVab at diagnosis, 2D/3D radiation technique, actual radiation dose of < 6600 cGy, and hospitalization during CRT were associated with poor OS, whereas baseline BMI of ≥23 kg/m^2^ and adjuvant chemotherapy were associated with longer OS.

**Table 3:** Univariate and multivariate analyses for overall survival

Variables	Univariate		Multivariate	
	Crude HR (95%CI)	***p*** value	Adjusted HR (95%CI)	***p*** value
Age ≥ 65 years	1.71 (1.24, 2.36)	0.001	1.6 (1.12, 2.3)	0.01
Male	0.78 (0.61, 1)	0.052	0.73 (0.54, 1)	0.052
Smoking	1.66 (1.32, 2.09)	< 0.001	1.01 (0.75, 1.36)	0.938
Stage at diagnosis				
I/II	Reference	1	Reference	1
III	1.45 (0.99, 2.14)	0.057	1.48 (0.98, 2.22)	0.061
IVab	3.4 (2.33, 4.95)	< 0.001	3.35 (2.26, 4.96)	< 0.001
BMI				
18.5–22.9	Reference	1	Reference	1
< 18.5	1.71 (1.18, 2.48)	0.005	1.49 (1, 2.22)	0.051
≥ 23	0.65 (0.51, 0.82)	< 0.001	0.68 (0.53, 0.87)	0.002
Radiation technique				
IMRT	Reference	1	Reference	1
2D/3D	2.05 (1.64, 2.57)	< 0.001	1.57 (1.18, 2.09)	0.002
RT delay	1.46 (1.14, 1.88)	0.003	1.08 (0.81, 1.45)	0.586
Actual dose of radiation (cGy)				
6600	Reference	1	Reference	1
< 6600	2.56 (1.56, 4.17)	< 0.001	1.89 (1.08, 3.33)	0.026
Cisplatin schedule during CRT				
Q1 week	Reference	1		
Q3 week	1.36 (0.78, 2.38)	0.284		
Cisplatin dose during CRT				
Intermediate	Reference	1	Reference	1
Low	1.46 (1.04, 2.06)	0.028	0.86 (0.58, 1.27)	0.444
High	1.36 (1.07, 1.72)	0.013	1.3 (0.99, 1.7)	0.057
Chemotherapy interruption during CRT	1.33 (1.07, 1.66)	0.011	1.33 (0.99, 1.78)	0.056
Chemotherapy delay during CRT	1.52 (1.18, 1.96)	0.001	0.93 (0.67, 1.3)	0.685
Hospitalization during CRT	2.39 (1.72, 3.32)	< 0.001	1.57 (1.08, 2.28)	0.019
Chemotherapy termination during CRT	1.17 (0.9, 1.52)	0.244		
Adjuvant chemotherapy	0.63 (0.46, 0.85)	0.002	0.59 (0.41, 0.84)	0.003
All cisplatin-related complications	1.44 (1.15, 1.79)	0.001	1.16 (0.89, 1.51)	0.274
AKD	1.21 (0.93, 1.58)	0.149		
AKI	1.91 (1.33, 2.74)	< 0.001		

*RT* radiotherapy, *CRT* chemoradiotherapy, *IMRT* intensity-modulated radiation therapy, *AKI* acute kidney injury, *AKD* acute kidney disease

## Discussion

Patients who received an intermediate CD of cisplatin (151–250 mg/m^2^; mean 207 mg/m^2^) achieved the highest survival and lowest overall complications and cisplatin-related toxicities. However, only univariate analysis showed that cisplatin CD during CRT associated with OS. This suggests that cisplatin CD was not an independent prognostic factor for OS in non-metastatic NPC. Therefore, the poorer survival in the low and high CD groups might have been affected by other related factors. Most of those who received a high CD of cisplatin (> 250 mg/m^2^; mean 287 mg/m^2^) were treated with the standard recommended CD of cisplatin based on several pivotal phase III studies [5–7]. However, the high CD patients included herein developed significant more complications and cisplatin-related toxicities during treatment than the other groups. Though incidences of AKI were comparable among these groups, the high CD group had significantly higher incidences of AKD, cisplatin-related hospitalization, all cisplatin-related toxicities, and CCr decline after completion of CRT (Table 2). In contrast, patients who received low cisplatin CD (≤150 mg/m^2^; mean 104 mg/m^2^) had poorer survival and more complications and cisplatin-related toxicities. Interestingly, none of the patients included herein satisfied the cisplatin ineligibility criteria for patients with head and neck cancer undergoing CRT in the Asia Pacific region [20]. However, some patients in this group could be considered high risk due to their age (> 70 years) and ECOG of 2. Moreover, majority of the patients in the low CD group could have been cisplatin ineligible in the real world given that half of them suffered from cisplatin-related toxicity, whereas two-third experienced interruptions during cisplatin treatment. In addition, the present study showed that patients who suffered from cisplatin-related toxicity had poor OS. Therefore, we hypothesized that low survival observed in high CD group could have been attributed to cisplatin-related complications, which interfered with other significant treatments factors, such as treatment interruption, hospitalization, RT dose, and delay. However, it remains inconclusive whether the low survival observed in the low CD group was indeed related to inadequate cisplatin CD during CRT or intolerability to cisplatin that might have affected the primary treatment, RT.

A post-hoc analysis of a Chinese prospective phase III study involving 298 locally advanced NPC patients who underwent CRT reported that a cisplatin CD of ≥240 mg/m^2^ was not an independent prognostic factor for OS [9]. A larger retrospective study on 549 patients with locally advanced NPC who underwent concurrent cisplatin and IMRT from the same group revealed similar results [12]. Another retrospective Chinese study including 491 patients with locally advanced NPC who received with cisplatin and IMRT [10] showed that those who received a low cisplatin CD (≤100 mg/m^2^) had poorer OS and DRFS than those who received intermediate (101 to ≤200 mg/m^2^) and high CDs (> 200 mg/m^2^). These results are similar to those presented herein given that our intermediate CD group received a mean cisplatin CD of 207 mg/m^2^. However, none of the previous studies included a high CD group that received close to the recommended cisplatin CD (280–300 mg/m^2^) to which we could compared [7, 9, 10, 12]. Moreover, none of these previous studies reported overall complications and cisplatin-related toxicities during CRT in each group.

Recently, induction chemotherapy with gemcitabine and cisplatin has prolonged OS and become the standard of care for treating locally advanced NPC [8]. Most patients (97%) completed three cycles of induction chemotherapy, wherein cisplatin CD was 240 mg/m^2^. During CRT, patients in the induction group received significantly less median dose intensity than the standard group (200 vs. 300 mg/m^2^; *p* < 0.001). A Chinese retrospective study showed no difference in OS among cisplatin CDs of more or less 160 mg/m^2^ during CRT for patients with locally advanced NPC receiving induction chemotherapy [16]. A larger study on 990 Chinese patients with NPC undergoing induction chemotherapy showed that a cisplatin CD of ≤100 mg/m^2^ was an independent prognostic factor for PFS and DRFS, but not for OS, among those who responded to induction chemotherapy [17]. Therefore, the optimal CD of cisplatin during CRT after completion of induction chemotherapy could perhaps be lower than that recommended [7, 8]. Most studies, including our study, have suggested that the lowest cisplatin CD during CRT (at least 150–200 mg/m^2^) might not affect survival [9, 10, 19, 20].

Interestingly, the effect of cisplatin CD on OS was not observed among patients who received IMRT but was pronounced among those who received non-IMRT. An important caveat to this study is its retrospective nature, which led to prognostically relevant baseline differences in radiation treatments received by patients. These often result from physicians’ selection bias and the availability of IMRT at the time of diagnosis. Notably, IMRT is considered a modern technique when compared to 2D or 3D radiotherapy, thus the majority of patients in this study were treated with non-IMRT techniques in the earlier years, in contrast to NPC patients from large cancer centers in Thailand, who are often treated with IMRT. Nonetheless, IMRT provides significantly better survival and lower serious toxicity compared to 2D and 3D techniques for the treatment of locally advanced NPC [21]. The effects of IMRT might override the benefit of adding it to concurrent chemotherapy for intermediate-risk (stage II and T3N0M0) NPC patients given that no improvement in OS had been observed [22, 23]. Thus, a similar phenomenon may be observed herein for the variation of cisplatin CD.

Our study contains several limitations, especially selection bias of the actual CD of cisplatin administration during CRT due to retrospective nature of the study. To our knowledge, no phase III study has evaluated the optimal cisplatin CD during CRT for locally advanced NPC. All retrospective studies, including the present study, contain limitations, particularly selection bias of treating physicians, due to their retrospective nature. Therefore, in clinical practice, patients with locally advanced NPC might be receiving CD of cisplatin as recommended by the standard guideline or pivotal phase III randomized studies [5–8]. This may lead to unnecessary complications and toxicities, especially from cisplatin [14, 15]. Moreover, these complications and toxicities may delay and/or affect radiation treatment, leading to poor survival. Thus, a randomized phase 3 study that would evaluate the optimal cisplatin CD during CRT for locally advanced NPC is warranted.

## Conclusions

Though intermediate cisplatin CD (151–250 mg/m^2^) during CRT was not an independent prognostic factor for OS, it allowed for minimal overall complications and cisplatin-related toxicities without compromising survivals. Moreover, cisplatin-related toxicities during CRT were associated with poor OS. Thus, an intermediate dose should be considered as the optimal cisplatin CD concurrent with radiation for non-metastatic NPC. Nonetheless, a randomized phase III study that would evaluate the optimal cisplatin CD during CRT for locally advanced NPC is warranted.

## Supplementary information

**Additional file 1: Supplement 1.** Cumulative dose of cisplatin (mg/m^2^) cutoff using the gold standard for death status. **Supplement 2.** Baseline characteristics of patients treated with different radiation technique. **Supplement 3.** Cisplatin-related toxicity during chemoradiotherapy in all patients. **Supplement 4.** Overall survival of patients who experienced cisplatin-related toxicities during chemoradiotherapy.

## Data Availability

Not applicable.

## Abbreviations

AKD: Acute kidney disease
AKI: Acute kidney injury
CCr: Creatinine clearance
CD: Cumulative dose
CI: Confidence intervals
CRT: Chemoradiotherapy
CT: Chemotherapy
DRFS: Distant recurrence-free survival
EBV: Epstein–Barr virus
GFR: Glomerular filtration rate
HR: Hazard ratios
IMRT: Intensity-modulated radiation therapy
LRFS: Locoregional recurrence-free survival
NPC: Nasopharyngeal carcinoma
OS: Overall survival
RFS: Recurrence-free survival
RT: Radiotherapy
RUN: Research University Network
