# Supplementary material for: Optimal cumulative dose of cisplatin for concurrent chemoradiotherapy among patients with non-metastatic nasopharyngeal carcinoma: a multicenter analysis in Thailand
Source: BMC Cancer. 2020 Jun 3;20:518. doi: 10.1186/s12885-020-07024-8 (PMC7271552; doi:10.1186/s12885-020-07024-8)

**Supplement 1.** Cumulative dose of cisplatin (mg/m^2^) cutoff using the gold standard for death status

| **Cutoff (≥) dose** | **TP** | **FP** | **FN** | **TN** | **Sen** | **Spec** | **PPV** | **NPV** | **Accuracy** | **LR+** | **LR-** | **p value** |
| --- | --- | --- | --- | --- | --- | --- | --- | --- | --- | --- | --- | --- |
| 38 | 313 | 465 | 0 | 1 | 100.0% | 0.2% | 40.2% | 100.0% | 40.3% | 1.00 | 0.00 | 0.412 |
| 50 | 313 | 462 | 0 | 4 | 100.0% | 0.9% | 40.4% | 100.0% | 40.7% | 1.01 | 0.00 | 0.100 |
| 68 | 312 | 462 | 1 | 4 | 99.7% | 0.9% | 40.3% | 80.0% | 40.6% | 1.01 | 0.37 | 0.356 |
| 78 | 310 | 457 | 3 | 9 | 99.0% | 1.9% | 40.4% | 75.0% | 40.9% | 1.01 | 0.50 | 0.280 |
| 83 | 301 | 454 | 12 | 12 | 96.2% | 2.6% | 39.9% | 50.0% | 40.2% | 0.99 | 1.49 | 0.319 |
| 88 | 301 | 453 | 12 | 13 | 96.2% | 2.8% | 39.9% | 52.0% | 40.3% | 0.99 | 1.37 | 0.418 |
| 95 | 299 | 452 | 14 | 14 | 95.5% | 3.0% | 39.8% | 50.0% | 40.2% | 0.98 | 1.49 | 0.280 |
| 103 | 279 | 430 | 34 | 36 | 89.1% | 7.7% | 39.4% | 51.4% | 40.4% | 0.97 | 1.41 | 0.133 |
| 108 | 279 | 429 | 34 | 37 | 89.1% | 7.9% | 39.4% | 52.1% | 40.6% | 0.97 | 1.37 | 0.165 |
| 115 | 279 | 428 | 34 | 38 | 89.1% | 8.2% | 39.5% | 52.8% | 40.7% | 0.97 | 1.33 | 0.201 |
| 128 | 278 | 425 | 35 | 41 | 88.8% | 8.8% | 39.5% | 53.9% | 40.9% | 0.97 | 1.27 | 0.272 |
| 138 | 277 | 425 | 36 | 41 | 88.5% | 8.8% | 39.5% | 53.2% | 40.8% | 0.97 | 1.31 | 0.215 |
| 143 | 275 | 424 | 38 | 42 | 87.9% | 9.0% | 39.3% | 52.5% | 40.7% | 0.97 | 1.35 | 0.159 |
| 148 | 275 | 423 | 38 | 43 | 87.9% | 9.2% | 39.4% | 53.1% | 40.8% | 0.97 | 1.32 | 0.192 |
| 153 | 270 | 412 | 43 | 54 | 86.3% | 11.6% | 39.6% | 55.7% | 41.6% | 0.98 | 1.19 | 0.373 |
| 158 | 269 | 409 | 44 | 57 | 85.9% | 12.2% | 39.7% | 56.4% | 41.8% | 0.98 | 1.15 | 0.457 |
| 163 | 260 | 397 | 53 | 69 | 83.1% | 14.8% | 39.6% | 56.6% | 42.2% | 0.98 | 1.14 | 0.423 |
| 168 | 260 | 395 | 53 | 71 | 83.1% | 15.2% | 39.7% | 57.3% | 42.5% | 0.98 | 1.11 | 0.526 |
| 173 | 259 | 391 | 54 | 75 | 82.7% | 16.1% | 39.8% | 58.1% | 42.9% | 0.99 | 1.07 | 0.670 |
| 178 | 255 | 390 | 58 | 76 | 81.5% | 16.3% | 39.5% | 56.7% | 42.5% | 0.97 | 1.14 | 0.421 |
| 185 | 247 | 364 | 66 | 102 | 78.9% | 21.9% | 40.4% | 60.7% | 44.8% | 1.01 | 0.96 | 0.790 |
| 193 | 231 | 344 | 82 | 122 | 73.8% | 26.2% | 40.2% | 59.8% | 45.3% | 1.00 | 1.00 | 0.996 |
| 198 | 230 | 342 | 83 | 124 | 73.5% | 26.6% | 40.2% | 59.9% | 45.4% | 1.00 | 1.00 | 0.977 |
| 203 | 174 | 256 | 139 | 210 | 55.6% | 45.1% | 40.5% | 60.2% | 49.3% | 1.01 | 0.99 | 0.857 |
| 208 | 174 | 255 | 139 | 211 | 55.6% | 45.3% | 40.6% | 60.3% | 49.4% | 1.02 | 0.98 | 0.811 |
| 213 | 169 | 254 | 144 | 212 | 54.0% | 45.5% | 40.0% | 59.6% | 48.9% | 0.99 | 1.01 | 0.888 |
| 216 | 169 | 253 | 144 | 213 | 54.0% | 45.7% | 40.0% | 59.7% | 49.0% | 0.99 | 1.01 | 0.935 |
| 219 | 169 | 252 | 144 | 214 | 54.0% | 45.9% | 40.1% | 59.8% | 49.2% | 1.00 | 1.00 | 0.982 |
| 223 | 169 | 250 | 144 | 216 | 54.0% | 46.4% | 40.3% | 60.0% | 49.4% | 1.01 | 0.99 | 0.924 |
| 228 | 159 | 215 | 154 | 251 | 50.8% | 53.9% | 42.5% | 62.0% | 52.6% | 1.10 | 0.91 | 0.202 |
| 233 | 155 | 207 | 158 | 259 | 49.5% | 55.6% | 42.8% | 62.1% | 53.1% | 1.11 | 0.91 | 0.162 |
| 238 | 150 | 203 | 163 | 263 | 47.9% | 56.4% | 42.5% | 61.7% | 53.0% | 1.10 | 0.92 | 0.231 |
| 243 | 124 | 154 | 189 | 312 | 39.6% | 67.0% | 44.6% | 62.3% | 56.0% | 1.20 | 0.90 | 0.061 |
| 246 | 122 | 151 | 191 | 315 | 39.0% | 67.6% | 44.7% | 62.3% | 56.1% | 1.20 | 0.90 | 0.059 |
| 247 | 122 | 150 | 191 | 316 | 39.0% | 67.8% | 44.9% | 62.3% | 56.2% | 1.21 | 0.90 | 0.051 |
| 249 | 122 | 149 | 191 | 317 | 39.0% | 68.0% | 45.0% | 62.4% | 56.4% | 1.22 | 0.90 | 0.044* |
| 253 | 119 | 145 | 194 | 321 | 38.0% | 68.9% | 45.1% | 62.3% | 56.5% | 1.22 | 0.90 | 0.046* |
| 258 | 111 | 144 | 202 | 322 | 35.5% | 69.1% | 43.5% | 61.5% | 55.6% | 1.15 | 0.93 | 0.183 |
| 263 | 101 | 131 | 212 | 335 | 32.3% | 71.9% | 43.5% | 61.2% | 56.0% | 1.15 | 0.94 | 0.214 |
| 266 | 101 | 128 | 212 | 338 | 32.3% | 72.5% | 44.1% | 61.5% | 56.4% | 1.17 | 0.93 | 0.149 |
| 268 | 100 | 128 | 213 | 338 | 31.9% | 72.5% | 43.9% | 61.3% | 56.2% | 1.16 | 0.94 | 0.178 |
| 273 | 94 | 121 | 219 | 345 | 30.0% | 74.0% | 43.7% | 61.2% | 56.4% | 1.16 | 0.95 | 0.213 |
| 278 | 94 | 119 | 219 | 347 | 30.0% | 74.5% | 44.1% | 61.3% | 56.6% | 1.18 | 0.94 | 0.168 |
| 283 | 71 | 81 | 242 | 385 | 22.7% | 82.6% | 46.7% | 61.4% | 58.5% | 1.31 | 0.94 | 0.067 |
| 288 | 70 | 79 | 243 | 387 | 22.4% | 83.0% | 47.0% | 61.4% | 58.7% | 1.32 | 0.93 | 0.060 |
| 295 | 62 | 68 | 251 | 398 | 19.8% | 85.4% | 47.7% | 61.3% | 59.1% | 1.36 | 0.94 | 0.056 |
| 310 | 0 | 3 | 313 | 463 | 0.0% | 99.4% | 0.0% | 59.7% | 59.4% | 0.00 | 1.01 | 0.155 |

**Supplement.2** Baseline characteristics of patients treated with different radiation technique

| **Patient characteristics**  **N (%)** | **2D**  **N=138 (%)** | **3D**  **N=111 (%)** | **IMRT**  **N=415 (%)** | **p value** |
| --- | --- | --- | --- | --- |
| Median age (range) years | 48 [18-77] | 49 [19-77] | 51 [16-78] |  |
| Age ≥ 65 years | 11 (7.4%) | 11 (9%) | 57 (12.1%) | 0.220 |
| Sex |  |  |  |  |
| Female | 46 (30.9%) | 36 (29.5%) | 154 (31.2%) | 0.934 |
| Male | 103 (69.1%) | 86 (70.5%) | 339 (68.8%) |  |
| Smoking status |  |  |  |  |
| Never | 53 (36.8%) | 58 (49.6%) | 341 (74.1%) | <0.001 |
| Ever | 91(63.2%) | 59 (50.4%) | 119 (25.9%) |  |
| ECOG status |  |  |  |  |
| 0–1 | 133 (95.7%) | 118 (98.3%) | 199 (99.5%) | 0.043 |
| ≥2 | 6 (4.3%) | 2 (1.7%) | 1 (0.5%) |  |
| Baseline BMI |  |  |  |  |
| <18.5 | 23 (15.9%) | 9 (7.4%) | 36 (7.3%) | 0.002 |
| 18.5–22.9 | 68 (46.9%) | 53 (44.8%) | 193 (39.1%) |  |
| ≥23 | 54 (37.2%) | 59 (48.8%) | 264 (53.5%) |  |
| WHO classification |  |  |  |  |
| I | 2 (1.3%) | 2 (1.7%) | 5 (1.1%) | 0.409 |
| II | 72 (48.3%) | 65 (53.7%) | 271 (57.3%) |  |
| III | 75 (50.3%) | 54 (44.6%) | 197 (41.6%) |  |
| T-stage |  |  |  |  |
| T1-2 | 79 (53%) | 58 (48.3%) | 251 (53.2%) | 0.627 |
| T3–4 | 70 (47%) | 62 (51.7%) | 221 (46.8%) |  |
| LN Stage |  |  |  |  |
| 0–1 | 45 (37.5%) | 39 (37.1%) | 157 (36.3%) | 0.922 |
| 2–3 | 75 (62.5%) | 66 (62.9%) | 282 (64.2%) |  |
| Stage at diagnosis |  |  |  |  |
| I | 0 (0%) | 0 (0%) | 2 (0.4%) | <0.001 |
| II | 30 (20.1%) | 19 (15.6%) | 77 (16.3%) |  |
| III | 46 (30.9%) | 56 (45.9%) | 242 (51.2%) |  |
| IVa | 32 (21.5%) | 30 (24.6%) | 117 (24.7%) |  |
| IVb | 41 (27.5%) | 17 (13.9%) | 35 (7.4%) |  |
| Any comorbidity | 25 (19.1%) | 20 (28.6%) | 131 (28%) | 0.111 |
| Cardiac | 2 (1.3%) | 2 (1.6%) | 14 (2.8%) | 0.487 |
| Diabetes | 9 (6%) | 5 (4.1%) | 51 (10.3%) | 0.042 |
| Hyperlipidemia | 2 (1.3%) | 2 (1.6%) | 39 (7.9%) | 0.001 |
| Hypertension | 12 (8.1%) | 12 (9.8%) | 98 (19.9%) | <0.001 |
| Kidney Disease | 0 (0%) | 1 (0.009%) | 1 (0.002%) | N/A |
| Mean Baseline CCr (±SD) | 83.6 ± 28 | 86.5 ± 21 | 96.8 ± 29 | <0.001 |
| Prophylactic feeding tube | 94 (63.1%) | 96 (78.7%) | 204 (41.4%) | <0.001 |
|  |  |  |  |  |
| Mean cumulative dose of cisplatin during CRT (mg/m^2^) (±SD) | 240.4±65.7 | 233.3±61.6 | 212.88±60 | <0.001 |
| Adjuvant chemotherapy regimen  Cisplatin-5FU  Carboplatin-5FU  No adjuvant chemotherapy | 111 (74.5%)  21 (14.1)  17 (11.4%) | 106 (86.9%)  9 (7.4%)  7 (5.7%) | 385 (78.1)  32 (6.5%)  76 (15.4) | 0.002 |

**Supplement.3** Cisplatin-related toxicity during chemoradiotherapy in all patients

| **Toxicity** | **N = 779 (%)** |
| --- | --- |
| AKD | 206 (26.4) |
| AKI | 54 (6.9) |
| Vomiting (grades 3–4) | 31 (4.0) |
| Electrolyte imbalance (grades 3–4) | 28 (3.6) |
| Infection (grades 3–4) | 26 (3.3) |
| Anemia (grades 3–4) | 16 (2.0) |
| Thrombocytopenia (grades 3–4) | 7 (1) |
| Ototoxicity | 3 (<1%) |
| Others | 5 (<1%) |

AKI, acute kidney injury; AKD, acute kidney disease

**Supplement 4.** Overall survival of patients who experienced cisplatin-related toxicities during chemoradiotherapy


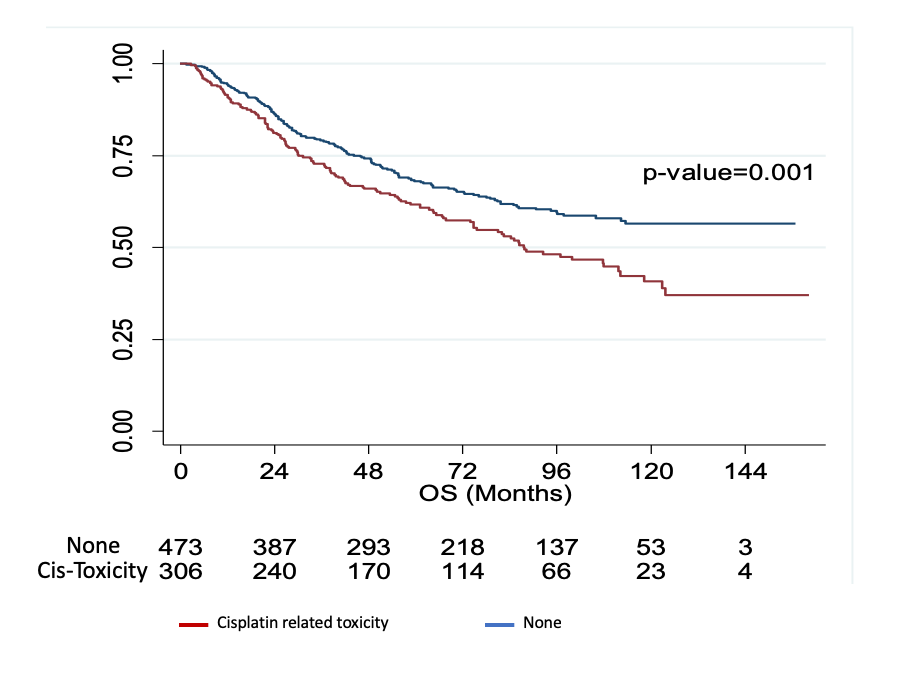

Supplement: Supplementary file 1 — Additional file 1: Supplement 1. Cumulative dose of cisplatin (mg/m2) cutoff using the gold standard for death status. Supplement 2. Baseline characteristics of patients treated with different radiation technique. Supplement 3. Cisplatin-related toxicity during chemoradiotherapy in all patients. Supplement 4. Overall survival of patients who experienced cisplatin-related toxicities during chemoradiotherapy. [file 12885_2020_7024_MOESM1_ESM.docx]
